# Impulsivity and sensitivity to reward as mediating factors of the negative relationship between emotional intelligence and health-related risk-taking: evidence from a sample of university students

**DOI:** 10.1186/s40359-023-01417-7

**Published:** 2023-11-09

**Authors:** Alberto Megías-Robles, María T. Sánchez-López, Raquel Gómez-Leal, Rosario Cabello, María José Gutiérrez-Cobo, Pablo Fernández-Berrocal

**Affiliations:** 1 Department of Basic Psychology, Faculty of Psychology, University of Málaga, Málaga, Spain; 2 Department of Developmental and Educational Psychology, Faculty of Psychology, University of Málaga, Málaga, Spain

**Keywords:** Risk-taking, Emotional intelligence, Health, Impulsivity, Sensitivity to reward

## Abstract

**Background:**

Better abilities in emotional intelligence (EI) have been linked to a decreased tendency to engage in health-related risk behaviour. However, the processes underlying this relationship are still unclear. The aim of this research was to examine the role of impulsivity and sensitivity to reward as mediating factors in the relationship between EI and health risk-taking.

**Methods:**

Two hundred and fifty participants (M_age_ = 23.60, age range = 18–59; SD = 6.67; 71.60% women) were assessed on ability EI levels, risk-taking in health contexts, impulsivity, and sensitivity to reward. Unlike previous studies in the literature, we employed a performance-based ability measure to assess EI (Mayer-Salovey-Caruso Emotional Intelligence Test, MSCEIT).

**Results:**

The results confirmed the negative relationship between EI and health risk-taking and revealed the existence of a significant negative indirect effect of EI on health-risk taking through various dimensions of impulsivity and sensitivity to reward. EI abilities —particularly the ability to manage emotions— were associated with lower levels of impulsivity under positive and negative emotional states, a better management of the tendency towards sensation seeking, and a decreased emotional reactivity to rewards.

**Conclusions:**

The present research provides a better understanding of the processes underlying the negative relationship between EI and health risk-taking. Our findings suggest that having higher levels of EI abilities would allow for a more objective evaluation of risk scenarios and a more appropriate and safer decision making through its influence on the levels of impulsivity and emotional reactivity to rewards. Practical implications, limitations, and future lines of research are discussed.

**Supplementary Information:**

The online version contains supplementary material available at 10.1186/s40359-023-01417-7.

## Background

Risk behaviour, within everyday naturalistic contexts, can be defined as those actions that lead to some probability of having negative outcomes, i.e., entail some chance of losing something of value or suffering harm [1–3]^1^. Behaviours such as drug initiation or use, unprotected sex, or reckless driving can take a heavy toll on our health, safety, and well-being. Many of these risky decisions are difficult to explain in terms of a rational and deliberative process of decision-making. In this regard, risk-taking models propose the involvement of heuristics and more automatic processes [4–6], with emotions having a decisive influence on our final actions [7–9].

The role that emotions play in risk behaviour has been demonstrated both at a behavioural and neural level [9–15]. When facing risky situations, our actions are often subject to time pressure and consequences with a strong emotional charge. It can therefore be difficult — if not impossible — to conduct a controlled and slow analytical appraisal of risk under such circumstances. Thus, in these types of scenarios, individuals neglect many of the analytical aspects involved in the decision-making process and make use of more automated and faster stimulus-response processes that are primarily guided by the affective values associated with that particular risk situation and its possible consequences [4, 8, 16, 17]. Likewise, numerous studies have also demonstrated the influence of incidental emotions on risk-taking, i.e., emotions elicited by external events unrelated to the risk situation [10, 11, 16, 18]. For example, Haase and Silbereisen [11] revealed how the induction of states of positive affect in a sample of adolescents and young adults led to a lower perception of risk regarding behaviours such as smoking, drinking alcohol, riding in a car with a drunk driver, or having unprotected sex. Finally, it is also worth noting that research studying the neural representation of risk behaviour has identified a brain network that comprises areas largely associated with emotional processing such as the anterior insula, anterior cingulate cortex, amygdala, and ventromedial prefrontal cortex [9, 12, 14].

Given the unequivocal evidence for the notion that people adapt their behaviour in risk situations through their emotions, it is expected that the individual abilities of perceiving, using, understanding, and managing emotions are key to explaining risk-taking tendencies. One particular construct that integrates all of these emotional abilities is emotional intelligence (EI). EI is defined as “the ability to perceive accurately, appraise, and express emotion; the ability to access and/or generate feelings when they facilitate thought; the ability to understand emotion and emotional knowledge; and the ability to regulate emotions to promote emotional and intellectual growth” [19, 20]. The intelligent use of emotions has been shown to have a positive impact on well-being and mental health [21, 22], whilst playing a protective role in maladaptive behaviours such as aggression and self-harm [23–25].

Previous research has also suggested the role of EI as a protective factor of engaging in risk behaviours that are dangerous to our health, such as unsafe sexual practices or substance abuse [26–31]. Although this negative relationship between EI and health risk-taking seems to be well documented, the mechanisms through which EI is associated with these behaviours remain poorly understood [32–34]. The present study seeks to shed further light on this issue and attempts to identify some of the factors mediating this relationship.

In the current literature on risk, two of the personality characteristics that have been most strongly linked to risk-taking are impulsivity and sensitivity to reward [35–41]. Reniers et al. [38] conducted a study on risk behaviour in adolescent and reported a path model through which they suggest that high impulsivity and sensitivity to reward are key personality variables that make this population prone to taking risks. For example, concerning impulsivity, Baltruschat et al. [35] revealed that an impulsive personality profile can predict risk proneness, and the brain functional connectivity patterns associated with these impulsivity traits differ according to whether or not the individuals are risk prone or not. In relation to sensitivity to reward, a literature review conducted by Scott-Parker and Weston [40] found that individuals with greater sensitivity to reward were consistently more likely to engage in risk behaviours such as drug use, dysfunctional drinking, or dysfunctional eating. Moreover, evidence from neuroimaging studies has demonstrated that risky behaviour is associated with increased activation of the ventro-medial prefrontal cortex and ventral striatum, brain areas commonly involved in the processing of rewards [42, 43].

As we will see below, both impulsivity and sensitivity to reward are constructs closely related to emotion. Current models explaining impulsivity consider this construct to be a multi-dimensional factor [44, 45]. The following five separable dimensions have been proposed [46]: positive and negative urgency (tendency to act rashly under conditions of positive or negative affect), sensation seeking (tendency to seek out new and exciting experiences that, on certain occasions, can be potentially dangerous), lack of premeditation (tendency to act without thinking about the consequences of an action before engaging in it), and lack of perseverance (inability to remain focused on a task). The dimensions of positive and negative urgency and sensation seeking are linked to motivation- and affect-driven aspects of impulsivity, while lack of premeditation and lack of perseverance are more closely linked to cognitive aspects of impulsivity [47–49]. Likewise, sensitivity to reward is characterized by an increased emotional reactivity to pleasurable and reinforcing outcomes, which can lead to a greater tendency to engage in approach behaviours aimed at achieving these appetitive outcomes, including risk behaviours [50–53].

Considering the influence of impulsivity and sensitivity to reward on the tendency to take risks, along with the prominent role played by emotion in these personality characteristics, we propose that impulsivity and sensitivity to reward can form part of the mediating mechanisms underlying the negative relationship between EI and health-related risk-taking. Given that EI involves a set of emotional abilities that are developed by the individual throughout the life cycle, we understand that these abilities, once acquired, can help reduce the original levels of impulsivity (particularly negative and positive urgency) and sensitivity to reward, which, in turn, are associated with a lower likelihood of engaging in health-related risk behaviour. Thus, the objectives of the current study were two-fold: (1) to confirm the negative relationship between EI and health-related risk-taking, and (2) to examine the possible indirect effect of EI on health-related risk-taking through the mediating role of impulsivity and sensitivity to reward. It is worth noting that, unlike the majority of previous studies on the relationship between EI and risk, we employed a performance-based ability measure to assess EI: the Mayer-Salovey-Caruso Emotional Intelligence Test (MSCEIT) [54]. This instrument allows us to obtain a total score of EI and a score for each of the four EI branches proposed in Mayer and Salovey’s model: perceiving, facilitating, understanding, and managing emotions [19, 20]. We believe that it is important to highlight the use of this test because previous studies in the literature have revealed a lack of a relationship between different EI models (ability vs. mixed and self-report vs. performance), supporting the notion that the performance-based ability model has greater convergent validity and a stronger capacity to predict general behaviour [55–58].

To address our aims, we tested the following hypotheses:

- H1. EI (and EI branches) is negatively associated with health-related risk-taking.
- H2. EI (and EI branches) is negatively related to impulsivity. We expected to find a strong relationship with those dimensions that are more closely linked to emotional processes (i.e., positive and negative urgency and sensation seeking).
- H3. EI (and EI branches) is negatively related to sensitivity to reward.
- H4. The dimensions of impulsivity and sensitivity to reward are positively associated with health risk-taking.
- H5. EI (and EI branches) is negatively and indirectly related to health risk-taking through the mediating role of impulsivity (particularly via positive and negative urgency and sensation seeking).
- H6. EI (and EI branches) is negatively and indirectly related to health risk taking through the mediating role of sensitivity to reward.
- H7. Finally, we were interested in identifying those direct and indirect effects that best predicted the relationship between EI (and EI branches) and health-related risk-taking (i.e., including all previous mediators in a single mediation model). We cannot stablish a clear hypothesis in relation to this issue, but we proposed a model that is particularly characterized by the dimensions of impulsivity that are most strongly linked to emotional factors, given their association with EI.

## Methods

### Participants

Two hundred and fifty participants voluntarily took part in the study. They were recruited by advertisements placed around the campus of the University of Malaga and on social networks and online platforms associated with this university. The mean age of the sample was 23.60 years (SD = 6.67; age range = 18–59), 71.60% of which were women (179 women and 71 men). Participants were treated in accordance with the Declaration of Helsinki and all of them signed an informed consent form assuring confidentiality and anonymity of the collected data [59]. The study was approved by The Research Ethics Committee of the University of Malaga (approval number: CEUMA 14-2019-H).

### Procedure and instruments

The participants were assessed on EI, levels of risk-taking in health contexts, impulsivity, and sensitivity to reward through the online platform LimeSurvey (http://limesurvey.org) using the following instruments.

Mayer-Salovey-Caruso Emotional Intelligence Test (MSCEIT) [54, 60]. The MSCEIT is a performance-based measure of ability EI. The instrument is composed of 141 items divided into four branches (dimensions): perceiving, facilitating, understanding, and managing emotions [19, 20]. The instrument provides a total EI score and individual scores for each branch. These scores are reported in a similar way to traditional cognitive intelligence tests, with an average score of 100 and a standard deviation of 15 [61]. The MSCEIT takes approximately 45 min to complete. In the current study we used the Spanish version of the MSCEIT [62], which has shown psychometric properties similar to the English version, with solid evidence of convergent and discriminant validity, and a high reliability (Cronbach’s α = 0.95) [60]. The internal consistency in our sample for MSCEIT total was α = 0.85; while for the MSCEIT branches these values ranged between 0.66 and 0.84.

Domain-specific risk-taking scale (DOSPERT-30) [63]. The DOSPERT-30 is a standardized self-report measure of risk behaviour. It consists of two parallel subscales, one assessing risk taking, i.e., the likelihood of engaging in a set of risky behaviours, and the other assessing risk perception, i.e., the level of perceived risk associated with those risk behaviours. Each subscale is composed of 30 items rated on a 7-point Likert scale ranging from 0 (Extremely Unlikely/Not at all Risky) to 6 (Extremely Likely/Extremely Risky). The risk situations presented in the questionnaire covers of five different life domains of health/safety, ethical, financial, recreational, and social (six items per domain). In the present study, we were specifically interested in the health/safety domain of the risk-taking subscale. Sample items of this domain include “Engaging in unprotected sex”, “Drinking heavily at a social function”, or “Driving a car without wearing a seat belt”. The total score was calculated by summing the responses to the six items corresponding to the health/safety domain (ranging from 0 to 42). We used the Spanish version of the questionnaire, which have shown adequate psychometric properties and a factorial structure similar to the English version [64]. In our sample the internal consistency for the health/safety domain was adequate (ordinal α = 0.77).

UPPS-P Impulsive behaviour scale, short Spanish version [45, 65]. The Spanish UPPS-P short version is a self-report instrument designed to assess impulsivity through the following five dimensions: negative urgency, positive urgency, lack of premeditation, lack of perseverance, and sensation seeking. The scale consists of 20 items (4 items per dimension). Items are rated on a 4-point Likert scale ranging from 1 (strongly agree) to 4 (strongly disagree). Some sample items include “When I am upset, I often act without thinking” or “My thinking is usually careful and purposeful”. The total score for each dimension was computed by summing the responses to the items corresponding to that dimension (ranging from 4 to 16). This scale has shown satisfactory internal consistency and external validity [65]. In our sample, internal consistency for the UPPS dimensions was adequate, ranging between ordinal α = 0.79 and 0.88.

Sensitivity to punishment and sensitivity to reward questionnaire-20 (SPSRQ–20) [66]. The SPSRQ–20 is a 20-item self-report instrument designed to assess levels of sensitivity to reward and punishment (10 items for each subscale). Responses are given on a dichotomous “yes-no” scale. Sample items include “Do you sometimes do things for quick gains?” (sensitivity to reward) or “Are you often afraid of new or unexpected situations?” (sensitivity to punishment). The scores are computed by adding the “yes” responses for each subscale (ranging from 0 to 10). The questionnaire has shown satisfactory psychometric properties [66]. For the purposes of this research, we were only interested in the sensitivity to reward subscale. Nonetheless, correlations between sensitivity to punishment and the rest of the study variables are shown in Table S1 for any readers who may be interested in this information (see online supplementary material). In our sample the internal consistency for the sensitivity to reward subscale was good (ordinal α = 0.82).

### Data analysis

First, descriptive analyses were carried out on the research variables. Second, given that the previous literature has suggested the existence of differences between men and women for both EI and risk behaviour [67–69], we decided to use Student t-tests to examine possible gender differences in order to include this factor, if significant, as a covariate in subsequent analyses. Third, to test H1, H2, H3, and H4, Pearson’s correlations were calculated between the study variables. Fourth, we conducted a stepwise multiple regression with the aim of identifying the dimensions of impulsivity and sensitivity to reward that are the strongest predictors of health-related risk-taking. The identification of these factors allowed us to analyse, in subsequent mediation analyses, those variables that had greater relevance as mediators. Fifth, and to address H5 and H6, simple mediation analyses were conducted to explore the mediating role of the dimensions of impulsivity and sensitivity to reward on the relationship between MSEIT total (also MSCEIT branches) and health-related risk-taking. As noted above, only significant predictors resulting from the previous stepwise multiple regression analysis were included as mediators. Finally, once the simple mediating effects had been established, and following the idea proposed in H7, we were interested in identifying those direct and indirect effects that better predicted the role of MSCEIT total (and MSCEIT branches) in health-related risk-taking. To this end, we tested a more complex path model that included, in a single model, all the direct and indirect effects that were found to be significant in the previous simple mediation analyses. For the case of MSCEIT branches, the four branches were entered together as predictors, and covariances between them were added to the model.

The descriptive analyses, t-tests, and Pearson’s correlations were conducted using SPSS version 24.0 (IBM Corporation, Armonk NY, USA). The alpha significance level was set at 0.05. Mediation and path analyses were conducted using IBM SPSS AMOS 21.0 software based on the maximum likelihood method. Indirect effects were estimated using the bias-corrected bootstrapping method (1,000 samples, 95% CI).

## Results

Descriptive statistics and the results of the t-tests for gender differences are shown in Table 1. Men, compared with women, showed higher scores on health risk-taking and sensitivity to reward (*p*s < 0.05) and lower scores on MSCEIT managing (*p* < .05). No other significant gender differences were observed.

**Table 1:** Descriptive statistics (mean [x̅] and standard deviation [SD]) and gender differences (determined by t-test) for the study variables

	x̅ (SD) Total sample	x̅ (SD) Men	x̅ (SD) Women	t	Cohen’s d
Risk-taking	20.17 (6.22)	21.75 (6.98)	19.55 (5.80)	2.55*	0.34
MSCEIT total	107.02 (8.60)	105.49 (8.92)	107.63 (8.41)	-1.78	0.25
MSCEIT perceiving	104.83 (11.05)	104.94 (10.24)	104.79 (11.38)	0.09	0.01
MSCEIT facilitating	101.10 (9.50)	100.34 (9.91)	101.40 (9.34)	-0.79	0.11
MSCEIT understanding	107.80 (10.13)	107.63 (10.69)	107.87 (9.93)	-0.16	0.02
MSCEIT managing	109.37 (11.51)	104.19 (13.06)	111.43 (10.16)	-4.67**	0.62
UPPS positive urgency	9.78 (2.31)	9.80 (2.42)	9.77 (2.27)	0.09	0.01
UPPS negative urgency	9.39 (3.04)	9.17 (3.23)	9.47 (2.96)	-0.72	0.10
UPPS lack of prem.	7.04 (2.16)	6.70 (2.15)	7.17 (2.16)	-1.55	0.22
UPPS lack of pers.	7.09 (2.36)	7.38 (2.39)	6.98 (2.35)	1.22	0.17
UPPS sensation seeking	10.65 (2.87)	11.10 (2.90)	10.47 (2.85)	1.57	0.22
Sensitivity to reward	3.78 (2.25)	4.51 (2.27)	3.49 (2.18)	3.31*	0.46

* *p* < .05, ** *p* < .01

Pearson’s correlation results (controlled for gender)^2^ are shown in Table 2. Focusing on the correlations of interest for H1, the results revealed a negative relationship between MSCEIT total and health-related risk-taking (*p* < .05). The MSCEIT branches of perceiving and managing were also negatively related to health-related risk-taking (*p*s < 0.05). With regard to H2 and H3, MSCEIT total was negatively related to all the dimensions of impulsivity (except UPPS lack of premeditation, *p* = .052) and to sensitivity to reward (*p* < .05). The MSCEIT branches showed the following relationships (*p*s < 0.05): MSCEIT perceiving was negatively related to UPPS positive urgency; MSCEIT facilitating was negatively related to UPPS positive urgency and UPPS sensation seeking; MSCEIT understanding was negatively related to UPPS positive urgency and UPPS negative urgency; and MSCEIT managing was negatively related to UPPS positive urgency, UPPS negative urgency, UPPS lack of premeditation, UPPS lack of perseverance, and sensitivity to reward. Finally, concerning H4, all the dimensions of impulsivity and sensitivity to reward were positively related to health-related risk-taking (*p*s < 0.05).

**Table 2:** Pearson’s correlation matrix for the study variables (controlling for gender)

	1	2	3	4	5	6	7	8	9	10	11	12
(1) Risk-taking	—											
(2) MSCEIT total	− 0.14*	—										
(3) MSCEIT perceiving	− 0.14*	0.77**	—									
(4) MSCEIT facilitating	− 0.08	0.67**	0.47**	—								
(5) MSCEIT understanding	− 0.01	0.58**	0.18*	0.14*	—							
(6) MSCEIT managing	− 0.13*	0.55**	0.16*	0.19*	0.22**	—						
(7) UPPS positive urgency	0.36**	− 0.27**	− 0.20*	− 0.14*	− 0.14*	− 0.21*	—					
(8) UPPS negative urgency	0.28**	− 0.14*	− 0.04	− 0.05	− 0.14*	− 0.16*	0.48**	—				
(9) UPPS lack of prem.	0.24**	− 0.12	− 0.04	− 0.04	− 0.10	− 0.14*	0.32**	0.36**	—			
(10) UPPS lack of pers.	0.17*	− 0.15*	− 0.10	− 0.12	− 0.04	− 0.13*	0.26**	0.26**	0.32**	—		
(11) UPPS sensation seeking	0.33**	− 0.18*	− 0.11	− 0.15*	− 0.12	− 0.10	0.33**	0.08	0.12	0.06	—	
(12) Sensitivity to reward	0.27**	− 0.16*	− 0.09	− 0.08	− 0.07	− 0.20*	0.34**	0.21**	0.11	0.07	0.29**	—

* *p* < .05, ** *p* < .01

Next, a stepwise multiple regression was carried out to identify those dimensions of impulsivity and sensitivity to reward that better predicted health-related risk-taking. Gender was also entered as predictor. This regression analysis revealed a final model accounting for 23% of the explained variance which involved UPPS positive urgency, UPPS negative urgency, UPPS sensation seeking, sensitivity to reward and gender as the predictors most strongly associated with risk-taking (see Table 3 for more details; Table S2 in online supplemental material presents a more detailed description of the included and excluded variables in the model).

To conduct the simple mediation analyses (H5 and H6), we focused only on those variables that were significant predictors of health-related risk-taking in the previous stepwise multiple regression analysis. Gender was controlled in all the mediation models. The models including MSCEIT total as predictor revealed a significant negative indirect effect of this variable on health-related risk-taking through the following mediators: UPPS positive urgency (standardized effect = -0.093, 95% CI [-0.123, -0.033]), UPPS negative urgency (standardized effect = -0.037, 95% CI [-0.058, -0.006]), UPPS sensation seeking (standardized effect = -0.056, 95% CI [-0.075, -0.014]), and sensitivity to reward (standardized effect = -0.040, 95% CI [-0.065, -0.008]). Interestingly, we did not observe significant direct effects of MSCEIT total on health-related risk-taking for any of these four mediators.

**Table 3:** Summary of the results for the final model resulting from the stepwise regression analysis

Criterion	Predictors	B	Std. error	*β*	t	*p*
Risk-taking	UPPS positive urgency	0.48	0.19	0.18	2.56	0.01
	UPPS sensation seeking	0.48	0.13	0.22	3.57	< 0.001
	Sensitivity to reward	0.31	0.17	0.11	1.80	0.07
	UPPS negative urgency	0.30	0.13	0.15	2.26	0.02
	Gender	-1.66	0.80	− 0.12	-2.08	0.04
	Constant	9.27	2.31		4.00	< 0.001

R^2^ = 0.23, *p* < .001

Regarding simple mediation analysis for the MSCEIT branches, MSCEIT perceiving showed a negative indirect effect on health-related risk-taking for the models in which the mediator was UPPS positive urgency (standardized effect = -0.069, 95% CI [-0.074, -0.016]) and UPPS sensation seeking (standardized effect = -0.033, 95% CI [-0.046, -0.001]), and a negative direct effect when the mediator was UPPS negative urgency (standardized effect = -0.125, 95% CI [-0.138, -0.014]), UPPS sensation seeking (standardized effect = -0.103, 95% CI [-0.125, -0.007]), and sensitivity to reward (standardized effect = -0.114, 95% CI [-0.0134, -0.006]). MSCEIT facilitating was indirectly and negatively related to risk-taking when the mediator was UPPS positive urgency (standardized effect = -0.049, 95% CI [-0.069, -0.003]) and UPPS sensation seeking (standardized effect = -0.047, 95% CI [-0.092, -0.008]), and none of the models showed significant direct effects. MSCEIT understanding was indirectly and negatively related to risk-taking when the mediator was UPPS positive urgency (standardized effect = -0.051, 95% CI [-0.070, -0.005]) and UPPS negative urgency (standardized effect = -0.040, 95% CI [-0.054, -0.003]), and no significant directs effects were revealed. MSCEIT managing was indirectly and negatively related to risk-taking through UPPS positive urgency (standardized effect = -0.075, 95% CI [-0.080, -0.013]), UPPS negative urgency (standardized effect = -0.044, 95% CI [-0.045, -0.006]), and sensitivity to reward (standardized effect = -0.052, 95% CI [-0.057, -0.009]), and no significant directs effects were revealed. Table S3 in online supplemental material summarizes the statistical results of the direct and indirect effects associated with each of the mediation models.

**Fig. 1:**
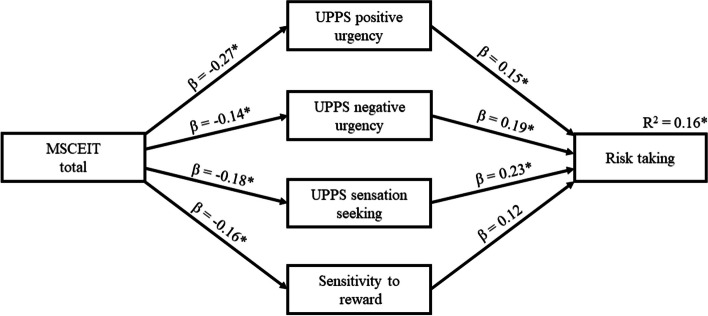
Representation of the mediation model including MSCEIT total as predictor, UPPS positive urgency, UPPS negative urgency, UPPS sensation seeking, and sensitivity to reward as mediators, and health risk-taking as criterion. Standardized path coefficients (β) and explained variance (R2) are displayed. Asterisks indicate statistical significance at *p* < .05

With the aim of identifying the mediating effects that best predict the role of MSCEIT total in health-related risk-taking (H7), we conducted an additional analysis based on a more complex mediation model that included the variables of UPPS positive urgency, UPPS negative urgency, UPPS sensation seeking, and sensitivity to reward as mediating variables in a single model. Only those direct and indirect effects that were found to be significant in the previous simple mediation models were included in the new model, and gender was entered as covariate. The resulting model is represented in Fig. 1. The path analysis revealed a significant indirect effect of MSCEIT total on health risk-taking through the four mediators together (standardized effect = -0.131, 95% CI [-0.146, -0.046]). Separate indirect effects for each mediator also remained significant (UPPS positive urgency: standardized effect = -0.050, 95% CI [-0.083, -0.009]; UPPS negative urgency: standardized effect = -0.022, 95% CI [-0.036, -0.001]; UPPS sensation seeking: standardized effect = -0.041, 95% CI [-0.056, -0.009]; sensitivity to reward: standardized effect = -0.019, 95% CI [-0.039, -0.001]). This model explained 16.2% of the variance in health risk-taking.

**Fig. 2:**
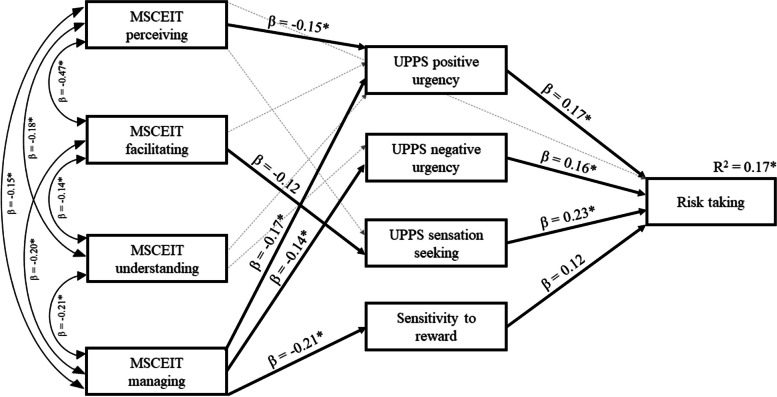
Representation of the mediation model including MSCEIT branches as predictors, UPPS positive urgency, UPPS negative urgency, UPPS sensation seeking, and sensitivity to reward as mediators, and health risk-taking as criterion. For ease of interpretation, paths associated with significant indirect effects are represented by bold lines along with standardized path coefficients. The rest of the non-significant paths entered into the model are represented by thin lines. Explained variance (R^2^) in health risk-taking is also indicated. Asterisks indicate statistical significance at *p* < .05

Finally, for the MSCEIT branches, we conducted a similar analytical procedure to that described above, including the four mediators in a single model. In this case, however, we also entered the four branches of the MSCEIT in the model. As described previously, the model only included those significant direct and indirect effects observed in the previous simple mediation analyses and gender was entered as covariate. The results revealed that MSCEIT managing was the most important branch predicting health risk-taking due to the indirect effects that this EI branch exerted through UPPS positive urgency, UPPS negative urgency, and sensitivity to reward (indirect effect of the three significant mediators together: standardized effect = -0.076, 95% CI [-0.078, -0.015]; UPPS positive urgency: standardized effect = -0.029, 95% CI [-0.049, -0.001]; UPPS negative urgency: standardized effect = -0.022, 95% CI [-0.031, -0.001]; sensitivity to reward: standardized effect = -0.024, 95% CI [-0.034, -0.001]). Moreover, MSCEIT perceiving was related to health risk-taking through UPPS positive urgency (standardized effect = -0.026, 95% CI [-0.042, -0.001]) and MSCEIT facilitating through UPPS sensation seeking (standardized effect = -0.028, 95% CI [-0.042, 0.001], *p* = .059, marginally significant). No significant direct effects were observed. The resulting model is displayed in Fig. 2. This model accounted for 16.6% of the explained variance in health risk-taking.

## Discussion

The current decision-making models explaining risk behaviour support the idea that emotion plays a major role on our actions when facing risk situations [4, 6, 8, 9]. In this regard, higher levels of EI, that is, better abilities in perceiving, facilitating, understanding, and managing emotions, have shown to be related to a decreased tendency to engage in health-related risk behaviours [26, 27, 29]. While this relationship has been well-documented, the processes underlying this connection still remain unclear. The aim of this research was to explore the potential role of impulsivity and sensitivity to reward as mediating factors in the relationship between EI and health risk-taking.

In accord with the previous literature, the results of the present study supported the existence of a negative relationship between MSCEIT total and health risk-taking, that is, the higher the levels of EI, the lower the likelihood of engaging in health risk behaviours [26–31]. Separate analysis of the four EI branches revealed that the MSCEIT perceiving and managing were the branches associated with health risk-taking. Thus, H1 was confirmed for EI total and for some of the EI branches. Similarly, higher levels of EI total were also associated with lower levels of impulsivity and sensitivity to reward, supporting H2 and H3. In this case, we observed that MSCEIT managing was the most significant branch. Finally, confirming H4, we observed that all dimensions of impulsivity and sensitivity to reward were positively related to health risk-taking.

In summary, these results suggest that EI could act as a possible protective factor in making risky decisions, decreasing the probability of engaging in behaviours that may threaten our own health and physical integrity, such as substance abuse, unsafe sexual practices or driving under the effects of alcohol. We also observed that an adequate level of EI — particularly in terms of the ability to manage emotions — is associated with reduced levels of impulsivity and sensitivity to reward. This relationship can be explained by current theoretical approaches in which appetitive emotion is assumed to be one of the main underlying factors of both impulsivity and sensitivity to reward [46, 52, 53]. At this point, given that impulsivity and sensitivity to reward have also been associated with a lower tendency to engage in health risk-taking, it makes sense to test H5, H6, and H7, in which we proposed that EI and health risk-taking are related through the mediating effect of these two factors.

Mediation analyses revealed a significant negative indirect effect of MSCEIT total on health risk-taking through the dimensions of impulsivity of positive and negative urgency and sensation seeking (H5) and through sensitivity to reward (H6). Hence, both H5 and H6 were supported for EI total. As for the individual branches of MSCEIT, we found a pattern of results in which not all the EI abilities were indirectly related to risk-taking. This pattern can be better observed from the results of H7 (i.e., direct and indirect effects that best predicted the relationship between EI and health-related risk-taking), which revealed that MSCEIT management was the most important branch for predicting health risk-taking through the mediating effect of positive and negative urgency and sensitivity to reward. MSCEIT perceiving and facilitating also showed an indirect effect on health risk-taking via positive urgency and sensation seeking, respectively. Here, it is worth noting that the dimensions of impulsivity that acted as mediators were those more closely linked to emotional processes.

These findings help to better understand the factors underlying the relationship between EI and risk-taking. Higher levels of EI, particularly regarding the ability to manage emotions, were associated with lower levels of impulsivity under positive and negative emotional states, lower tendency towards sensation seeking, and lower sensitivity to reward. These personality characteristics would allow individuals to reduce the tendency to be guided by reinforcing and emotionally driven behaviours, which could lead to a more objective assessment of risk scenarios, enabling more appropriate and safer decision making in terms of health preservation. For example, individuals characterized by a high impulsivity and reactivity to reward would tend to assign greater value to the benefits of risk behaviour than to the costs, making such behaviour more attractive to these individuals [39]. However, of these individuals, those who have good EI abilities, compared with those with low EI, would have a greater capacity for refusing to engage in risk behaviours such as unsafe sexual activities or drug consumption, even when their level of emotional arousal is high. Whilst more research is needed to support this idea, we propose that these individuals would be better able to identify and manage the emotion urging them to act and would thus be able to anticipate the possible consequences of their actions, making a more objective appraisal of the benefits and costs involved.

The practical implications of this work seem evident, since health-related risk behaviour is associated with a multitude of adverse consequences, such as medical problems, physical injuries, fatalities, and economic and social problems. The development and implementation of EI training programmes focused on enhancing emotional abilities, such as *RULER* or *INTEMO+* [70, 71], could yield significant advantages in reducing the prevalence of risk-taking in society. These programmes have the potential to equip individuals with improved emotional management skills, enabling them to effectively handle emotions linked to impulsive behaviour (positive and negative urges), sensation-seeking, or sensitivity to value of the rewards, all of which are factors that have been confirmed to be related to increased risk-taking in the present study. Moreover, we should not lose sight of the implications of working with EI in adolescents, a population that is over-represented in risk-taking and characterized by high levels of impulsiveness and sensitivity to reward [38]. The prevention of health risk behaviours in this critical lifecycle stage must be a priority for public health and school system policies.

Finally, some limitations of the study should be considered. First, it is important to emphasize that the nature of our study is correlational. Therefore, experimental studies must be conducted to establish causality and confirm the protective role of EI in risk-taking. Second, future studies should also replicate and extend our results to the general population including a community sample characterized by a more balanced gender and age distribution. Moreover, these participants should be selected by a random sampling procedure. Third, another issue to address is that the variables of risk-taking, impulsivity, and sensitivity to reward were assessed through self-report questionnaires. The responses to these types of questionnaires may be susceptible to social desirability bias and subjective perceptions, which sometimes do not correspond to reality. Further studies should be based, when possible, on performance measures that allow for more objective assessments. Fourth, it would be interesting to focus on more concrete and representative situations of risk with the aim of reducing bias and facilitating memory retrieval, using, for example, questions such as “how often did you drive drunk last year when you went out to have fun?”. Finally, we also recommend that future research examines the particular regulatory strategies by which impulsivity and sensitivity to reward are brought under control.

In conclusion, the present research provides evidence to support the existence of a negative relationship between EI and health risk-taking. Importantly, the emotional components of impulsivity and the levels of sensitivity to reward have shown to be among the mediating factors underlying this relationship. According to these results, we suggest that having higher EI abilities, particularly the ability to manage emotions, would allow us to control our impulsivity associated with positive and negative emotional states, to manage the tendency toward seeking new and exciting sensations, and to reduce our emotional reactivity to rewards. In turn, this better control of both impulsivity and the approach behaviours toward appetitive outcomes would allow us to make a better assessment of the cost and benefits of our actions, decreasing the tendency towards risk-taking and leading to more adaptive behaviour in risk contexts. Further experimental studies are needed to confirm this protective role of EI in health risk-taking, and if this is confirmed, our findings would have important implications at a public health level. Appropriate training in EI abilities could translate into a decrease in health-related risk behaviours and, in turn, mitigate the harmful consequences that many of these behaviours have for our lives.

### Supplementary Information

**Additional file 1: Table S1. **Pearson’s correlation matrix of the study variables (without controlling for gender). **Table S2.** Included and excluded variables in the final stepwise regression model identifying those dimensions of impulsivity and sensitivity to reward that better predicted health related risk-taking (gender was also entered as predictor). **Table S3.** Summary of results for the simple mediation analyses. Direct and indirect effects are presented for each model, which included MSCEIT total or MSCEIT branches as predictors of health risk-taking through the mediating effect of UPPS positive urgency, UPPS negative urgency, UPPS sensation seeking, or sensitivity to reward

## Data Availability

All data generated or analysed during this study are included in the supplementary material.

## Footnotes

1 In the field of economics, unlike “more naturalistic” risk-taking scenarios, risk behaviour is defined as the variance in the probability of outcomes more than the exposure to possible negative outcomes [2]

2 In case any reader is interested in the Pearson’s correlation results obtained without controlling for gender, we have included these in Table S1 in the online supplementary material

## References

[1] Fischhoff B, Kadvany J (2011). Risk: a very short introduction.

[2] Schonberg T, Fox CR, Poldrack RA (2011). Mind the gap: bridging economic and naturalistic risk-taking with cognitive neuroscience. Trends Cogn Sci.

[3] Yates JF, Stone ER, Yates JF (1992). The risk construct. Wiley series in human performance and cognition risk-taking behavior.

[4] Megías A, Navas JF, Petrova D, Cándido A, Maldonado A, Garcia-Retamero R (2015). Neural mechanisms underlying urgent and evaluative behaviors: an fMRI study on the interaction of automatic and controlled processes. Hum Brain Mapp.

[5] Reyna VF (2004). How people make decisions that involve risk. Curr Dir Psychol Sci.

[6] Slovic P, Finucane ML, Peters E, MacGregor DG (2004). Risk as analysis and risk as feelings: some thoughts about affect, reason, risk, and rationality. Risk Anal.

[7] Figner B, Mackinlay RJ, Wilkening F, Weber EU (2009). Affective and deliberative processes in risky choice: age differences in risk taking in the Columbia Card Task. J Exp Psychol Learn Mem Cogn.

[8] Loewenstein GF, Weber EU, Hsee CK, Welch N (2001). Risk as feelings. Psychol Bull.

[9] Mohr PN, Biele G, Heekeren HR (2010). Neural processing of risk. J Neurosci.

[10] Ferrer RA, Taber JM, Sheeran P, Bryan AD, Cameron LD, Peters E (2020). The role of incidental affective states in appetitive risk behavior: a meta-analysis. Heal Psychol.

[11] Haase CM, Silbereisen RK (2011). Effects of positive affect on risk perceptions in adolescence and young adulthood. J Adolesc.

[12] Megías A, Cándido A, Maldonado A, Catena A (2018). Neural correlates of risk perception as a function of risk level: an approach to the study of risk through a daily life task. Neuropsychologia.

[13] Rivers SE, Reyna VF, Mills B (2008). Risk taking under the influence: a fuzzy-trace theory of emotion in adolescence. Dev Rev.

[14] Vorhold V (2008). The neuronal substrate of risky choice: an insight into the contributions of neuroimaging to the understanding of theories on decision making under risk. Ann N Y Acad Sci.

[15] Zaleskiewicz T, Traczyk J, Sobkow A, Fulawka K, Megías-Robles A. Visualizing risky behaviors induces a stronger neural response in brain areas responsible for mental imagery and emotions than visualizing neutral behaviors. Res Sq. 2022;17:1–26. 10.3389/fnhum.2023.1207364 PMC10546025 37795209

[16] Megías A, Maldonado A, Cándido A, Catena A (2011). Emotional modulation of urgent and evaluative behaviors in risky driving scenarios. Accid Anal Prev.

[17] Slovic P, Finucane ML, Peters E, MacGregor DG (2007). The affect heuristic. Eur J Oper Res.

[18] Megías A, Di Stasi LL, Maldonado A, Catena A, Cándido A (2014). Emotion-laden stimuli influence our reactions to traffic lights. Transp Res Part F Traffic Psychol Behav.

[19] Mayer JD, Salovey P, Salovey P, Sluyter DJ (1997). What is emotional intelligence?. Emotional development and emotional intelligence: Educational implications.

[20] Mayer JD, Caruso DR, Salovey P (2016). The ability model of emotional intelligence: principles and updates. Emot Rev.

[21] Martins A, Ramalho N, Morin E (2010). A comprehensive meta-analysis of the relationship between Emotional Intelligence and health. Pers Individ Dif.

[22] Sánchez-Álvarez N, Extremera N, Fernández-Berrocal P (2016). The relation between emotional intelligence and subjective well-being: a meta-analytic investigation. J Posit Psychol.

[23] Megías A, Gómez-Leal R, Gutiérrez-Cobo MJ, Cabello R, Fernández-Berrocal P (2018). The relationship between aggression and ability emotional intelligence: the role of negative affect. Psychiatry Res.

[24] Mikolajczak M, Petrides KV, Hurry J (2009). Adolescents choosing self-harm as an emotion regulation strategy: the protective role of trait emotional intelligence. Br J Clin Psychol.

[25] Vega A, Cabello R, Megías-Robles A, Gómez-Leal R, Fernández-Berrocal P (2021). Emotional intelligence and aggressive behaviors in adolescents: a systematic review and meta-analysis. Trauma Violence Abus.

[26] Fernández-Abascal EG, Martín-Díaz MD (2015). Dimensions of emotional intelligence related to physical and mental health and to health behaviors. Front Psychol.

[27] Lana A, Baizán EM, Faya-Ornia G, López ML (2015). Emotional intelligence and health risk behaviors in nursing students. J Nurs Educ.

[28] Lando-King E, McRee AL, Gower AL, Shlafer RJ, McMorris BJ, Pettingell S (2015). Relationships between social-emotional intelligence and sexual risk behaviors in adolescent girls. J Sex Res.

[29] Rivers SE, Brackett MA, Omori M, Sickler C, Bertoli MC, Salovey P (2013). Emotion skills as a protective factor for risky behaviors among college students. J Coll Stud Dev.

[30] Zavala MA, López I (2012). Adolescentes en situación de riesgo psicosocial: ¿qué papel juega la inteligencia emocional? [Adolescents at psychosocial risk: what role does emotional intelligence play?]. Behav Psychol Conduct.

[31] Sánchez-López MT, Megías-Robles A, Gómez-Leal R, Gutiérrez-Cobo MJ, Fernández-Berrocal P (2018). Relación entre La Inteligencia Emocional Percibida Y El Comportamiento De Riesgo en El ámbito De La Salud. Escritos Psicol / Psychol Writings.

[32] Sánchez-López MT, Fernández-Berrocal P, Gómez-Leal R, Megías-Robles A (2022). Evidence on the relationship between emotional intelligence and risk behaviour: a systematic and meta-analytic review. Front Psychol.

[33] Megías-Robles A, Sánchez-López MT, Fernández-Berrocal P (2022). The relationship between self-reported ability emotional intelligence and risky driving behaviour: consequences for Accident and traffic ticket rate. Accid Anal Prev.

[34] Anwar T, Fatima I, Malik JA (2016). Risk factors of health risk behaviors in intermediate students. Pakistan J Psychol Res.

[35] Baltruschat S, Cándido A, Megías A, Maldonado A, Catena A. Risk proneness modulates the impact of impulsivity on brain functional connectivity. Hum Brain Mapp. 2020;41(4):943–51. 10.1002/hbm.24851 PMC7267946 31691415

[36] Donohew L, Zimmerman R, Cupp PS, Novak S, Colon S, Abell R (2000). Sensation seeking, impulsive decision-making, and risky sex: implications for risk-taking and design of interventions. Pers Individ Dif.

[37] Duell N, Steinberg L, Chein J, Al-Hassan SM, Bacchini D, Lei C (2016). Interaction of reward seeking and self-regulation in the prediction of risk taking: a cross-national test of the dual systems model. Dev Psychol.

[38] Reniers RL, Murphy L, Lin A, Bartolomé SP, Wood SJ (2016). Risk perception and risk-taking behaviour during adolescence: the influence of personality and gender. PLoS ONE.

[39] Reyna VF, Farley F (2006). Risk and rationality in adolescent decision making: implications for theory, practice, and public policy. Psychol Sci Public Interes.

[40] Scott-Parker B, Weston L. Sensitivity to reward and risky driving, risky decision making, and risky health behaviour: a literature review. Transp Res part F Traffic Psychol Behav. 2017;49:93–109.

[41] Zuckerman M, Kuhlman DM (2000). Personality and risk-taking: common biosocial factors. J Pers.

[42] Galvan A, Hare TA, Parra CE, Penn J, Voss H, Glover G (2006). Earlier development of the accumbens relative to orbitofrontal cortex might underlie risk-taking behavior in adolescents. J Neurosci.

[43] Van Leijenhorst L, Moor BG, de Macks ZAO, Rombouts SA, Westenberg PM, Crone EA (2010). Adolescent risky decision-making: neurocognitive development of reward and control regions. NeuroImage.

[44] Cyders MA, Smith GT, Spillane NS, Fischer S, Annus AM, Peterson C (2007). Integration of impulsivity and positive mood to predict risky behavior: development and validation of a measure of positive urgency. Psychol Assess.

[45] Whiteside SP, Lynam DR (2001). The five factor model and impulsivity: using a structural model of personality to understand impulsivity. Pers Individ Dif.

[46] Cyders MA, Littlefield AK, Coffey S, Karyadi KA (2014). Examination of a short English version of the UPPS-P Impulsive Behavior Scale. Addict Behav.

[47] Del Prete F, Steward T, Navas JF, Fernández-Aranda F, Jiménez-Murcia S, Oei TPS (2017). The role of affect-driven impulsivity in gambling cognitions: a convenience-sample study with a Spanish version of the Gambling-related cognitions Scale. J Behav Addict.

[48] Knezevic-Budisin B, Pedden V, White A, Miller CJ, Hoaken PNS (2015). A multifactorial conceptualization of impulsivity: implications for research and clinical practice. J Individ Differ.

[49] Sharma L, Markon KE, Clark LA (2014). Toward a theory of distinct types of impulsive behaviors: a meta-analysis of self-report and behavioral measures. Psychol Bull.

[50] Carver CS, White TL (1994). Behavioral inhibition, behavioral activation, and affective responses to impending reward and punishment: the BIS/BAS scales. J Pers Soc Psychol.

[51] Gilbert KE, Nolen-Hoeksema S, Gruber J (2016). I don’t want to come back down: undoing versus maintaining of reward recovery in older adolescents. Emotion.

[52] Pickering A, Gray JA. Dopamine, appetitive reinforcement, and the neuropsychology of human learning: an individual differences approach. Adv Individ Differ Res. 2001:113–49.

[53] Torrubia R, Avila C, Moltó J, Caseras X (2001). The sensitivity to punishment and sensitivity to reward questionnaire (SPSRQ) as a measure of Gray’s anxiety and impulsivity dimensions. Pers Individ Dif.

[54] Mayer JD, Salovey P, Caruso D (2002). Mayer-Salovey-Caruso Emotional Intelligence Test (MSCEIT) user’s Manual.

[55] Gutiérrez-Cobo MJ, Cabello R, Fernández-Berrocal P (2016). The relationship between emotional intelligence and cool and hot cognitive processes: a systematic review. Front Behav Neurosci.

[56] Joseph DL, Newman DA (2010). Emotional intelligence: an integrative meta-analysis and cascading model. J Appl Psychol.

[57] Webb CA, Schwab ZJ, Weber M, Del Donno S, Kipman M, Weiner MR (2013). Convergent and divergent validity of integrative versus mixed model measures of emotional intelligence. Intelligence.

[58] Brackett MA, Rivers SE, Shiffman S, Lerner N, Salovey P (2006). Relating emotional abilities to social functioning: a comparison of self-report and performance measures of emotional intelligence. J Pers Soc Psychol.

[59] World Medical Association (2009). Declaration of Helsinki: ethical principles for medical research involving human subjects.

[60] Sánchez-García M, Extremera N, Fernández-Berrocal P (2016). The factor structure and psychometric properties of the Spanish version of the Mayer-Salovey-Caruso Emotional Intelligence Test. Psychol Assess.

[61] Mayer J, Saloyev P, Caruso D (2002). The Mayer-Salovey-Caruso Emotional Intelligence Test (MSCEIT).

[62] Extremera N, Fernández-Berrocal P, Salovey P (2006). Spanish version of the Mayer-Salovey-Caruso Emotional Intelligence Test (MSCEIT). Version 2.0: reliabilities, age and gender differences. Psicothema.

[63] Blais AR, Weber EU (2006). A domain-specific risk-taking (DOSPERT) scale for adult populations. Judgm Decis Mak.

[64] Lozano LM, Megías A, Catena A, Perales JC, Baltruschat S, Cándido A (2017). Spanish validation of the domain-specific risk-taking (DOSPERT-30) scale. Psicothema.

[65] Cándido A, Orduña E, Perales JC, Verdejo-García A, Billieux J (2012). Validation of a short Spanish version of the UPPS-P impulsive behaviour scale. Trastor Adict.

[66] Aluja A, Blanch A (2011). Neuropsychological behavioral inhibition system (BIS) and behavioral approach system (BAS) assessment: a shortened sensitivity to punishment and sensitivity to reward questionnaire version (SPSRQ-20). J Pers Assess.

[67] Cabello R, Sorrel MA, Fernández-Pinto I, Extremera N, Fernández-Berrocal P (2016). Age and gender differences in ability emotional intelligence in adults: a cross-sectional study. Dev Psychol.

[68] Kritsotakis G, Psarrou M, Vassilaki M, Androulaki Z, Philalithis AE (2016). Gender differences in the prevalence and clustering of multiple health risk behaviours in young adults. J Adv Nurs.

[69] Peltzera K, Pengpida S (2015). Gender differences in health risk behaviour among university students: an international study. Gend Behav.

[70] Rivers SE, Brackett MA (2011). Achieving standards in the English language arts (and more) using the RULER approach to social and emotional learning. Read Writ Q.

[71] Cabello R, Castillo R, Rueda P, Fernández-Berrocal P. Programa INTEMO+. Mejorar la inteligencia Emocional de los adolescentes. Pirámide: 2016.

