# Supplementary material for: Impulsivity and sensitivity to reward as mediating factors of the negative relationship between emotional intelligence and health-related risk-taking: evidence from a sample of university students
Source: BMC Psychol. 2023 Nov 9;11:386. doi: 10.1186/s40359-023-01417-7 (PMC10636922; doi:10.1186/s40359-023-01417-7)
Supplement: Supplementary file 1 — Additional file 1: Table S1. Pearson’s correlation matrix of the study variables (without controlling for gender). Table S2. Included and excluded variables in the final stepwise regression model identifying those dimensions of impulsivity and sensitivity to reward that better predicted health related risk-taking (gender was also entered as predictor). Table S3. Summary of results for the simple mediation analyses. Direct and indirect effects are presented for each model, which included MSCEIT total or MSCEIT branches as predictors of health risk-taking through the mediating effect of UPPS positive urgency, UPPS negative urgency, UPPS sensation seeking, or sensitivity to reward [file 40359_2023_1417_MOESM1_ESM.docx]

**Table S1.** Pearson’s correlation matrix of the study variables (without controlling for gender)

|  | 1 | 2 | 3 | 4 | 5 | 6 | 7 | 8 | 9 | 10 | 11 | 12 |
| --- | --- | --- | --- | --- | --- | --- | --- | --- | --- | --- | --- | --- |
| (1) Risk taking | — |  |  |  |  |  |  |  |  |  |  |  |
| (2) MSCEIT total | -.16* | — |  |  |  |  |  |  |  |  |  |  |
| (3) MSCEIT perceiving | -.14* | .77** | — |  |  |  |  |  |  |  |  |  |
| (4) MSCEIT facilitating | -.08 | .67** | .47** | — |  |  |  |  |  |  |  |  |
| (5) MSCEIT understanding | -.01 | .58** | .18* | .14* | — |  |  |  |  |  |  |  |
| (6) MSCEIT managing | -.17* | .55** | .15* | .20* | .21* | — |  |  |  |  |  |  |
| (7) UPPS positive urgency | .36** | -.27** | -.20* | -.14* | -.14* | -.20* | — |  |  |  |  |  |
| (8) UPPS negative urgency | .26** | -.14* | -.04 | -.05 | -.14* | -.14* | .48** | — |  |  |  |  |
| (9) UPPS lack of prem. | .22* | -.10 | -.04 | -.04 | -.10 | -.10 | .31** | .37** | — |  |  |  |
| (10) UPPS lack of pers. | .18* | -.15* | -.10 | -.13* | -.04 | -.14* | .26** | .26** | .31** | — |  |  |
| (11) UPPS sensation seeking | .34** | -.19* | -.11 | -.15* | -.12* | -.13* | .33** | .08 | .11 | .07 | — |  |
| (12) Sensitivity to reward | .29** | -.18* | -.08 | -.09 | -.08 | -.25** | .33** | .19** | .09 | .08 | .30** | — |
| (13) Sensitivity to punishment | .01 | -.04 | -.03 | -.10 | .03 | -.01 | .10 | .27** | -.02 | .23** | -.28** | -.23* |
| * *p* < .05, ** *p* < .01 | | | | | | | | | | | | |

**Table S2.** Included and excluded variables in the final stepwise regression model identifying those dimensions of impulsivity and sensitivity to reward that better predicted health related risk-taking (gender was also entered as predictor).

| Included variables | | | | |
| --- | --- | --- | --- | --- |
| Criterion | Predictors | *β* | t | *p* |
| Risk-taking | UPPS positive urgency | .18 | 2.56 | .01 |
|  | UPPS sensation seeking | .22 | 3.57 | < .001 |
|  | Sensitivity to reward | .11 | 1.80 | .07 |
|  | UPPS negative urgency | .15 | 2.26 | .02 |
|  | Gender | -.12 | -2.08 | .04 |
|  | Constant |  | 4.00 | < .001 |
| Excluded variables | | | | |
| Criterion | Predictors | *β* | t | *p* |
| Risk-taking | UPPS lack of prem. | .10 | 1.70 | .09 |
|  | UPPS lack of pers. | .07 | 1.15 | .25 |
| R^2^ = 0.23, *p* < 0.001 | | | | |

**Table S3.** Summary of results for the simple mediation analyses. Direct and indirect effects are presented for each model, which included MSCEIT total or MSCEIT branches as predictors of health risk-taking through the mediating effect of UPPS positive urgency, UPPS negative urgency, UPPS sensation seeking, or sensitivity to reward.

| Predictor | Mediator | Effect | Unstandardized coeff. | Standard errors | Standardized coeff. | 95% CI  [lower, upper] | *p* |
| --- | --- | --- | --- | --- | --- | --- | --- |
| MSCEIT total | UPPS positive urgency  R^2^ = 0.16, *p* < .001 | Indirect effect | -0.067 | 0.022 | -0.093 | [-0.123, -0.033] | 0.001 |
|  |  | Direct effect | -0.033 | 0.044 | -0.046 | [-0.123, 0.056] | 0.439 |
|  | UPPS negative urgency  R^2^ = 0.11, *p* = 0.007 | Indirect effect | -0.027 | 0.013 | -0.037 | [-0.058, -0.006] | 0.012 |
|  |  | Direct effect | -0.074 | 0.043 | -0.102 | [-0.166, 0.008] | 0.075 |
|  | UPPS sensation seeking R^2^ = 0.14, *p* = 0.007 | Indirect effect | -0.040 | 0.015 | -0.056 | [-0.075, -0.014] | 0.003 |
|  |  | Direct effect | -0.060 | 0.041 | -0.083 | [-0.149, 0.015] | 0.118 |
|  | Sensitivity to reward  R^2^ = 0.10, *p* = 0.006 | Indirect effect | -0.029 | 0.014 | -0.040 | [-0.065, -0.008] | 0.006 |
|  |  | Direct effect | -0.071 | 0.042 | -0.098 | [-0.163, 0.012] | 0.089 |
|  |  |  |  |  |  |  |  |
| MSCEIT perceiving | UPPS positive urgency  R^2^ = 0.16, *p* = 0.006 | Indirect effect | -0.039 | 0.015 | -0.069 | [-0.074, -0.016] | 0.001 |
|  |  | Direct effect | -0.037 | 0.055 | -0.066 | [-0.102, 0.020] | 0.217 |
|  | UPPS negative urgency  R^2^ = 0.12, *p* = 0.007 | Indirect effect | -0.006 | 0.010 | -0.011 | [-0.026, 0.013] | 0.496 |
|  |  | Direct effect | -0.070 | 0.031 | -0.125 | [-0.138, -0.014] | 0.010 |
|  | UPPS sensation seeking R^2^ = 0.14, *p* = 0.007 | Indirect effect | -0.019 | 0.011 | -0.033 | [-0.046, -0.001] | 0.042 |
|  |  | Direct effect | -0.058 | 0.030 | -0.103 | [-0.125, -0.007] | 0.030 |
|  | Sensitivity to reward  R^2^ = 0.11, *p* = 0.006 | Indirect effect | -0.012 | 0.010 | -0.022 | [-0.035, 0.005] | 0.175 |
|  |  | Direct effect | -0.064 | 0.053 | -0.114 | [-0.134, -0.006] | 0.028 |
|  |  |  |  |  |  |  |  |
| MSCEIT facilitating | UPPS positive urgency  R^2^ = 0.15, *p* = 0.008 | Indirect effect | -0.032 | 0.017 | -0.049 | [-0.069, -0.003] | 0.027 |
|  |  | Direct effect | -0.017 | 0.038 | -0.026 | [-0.090, 0.057] | 0.694 |
|  | UPPS negative urgency  R^2^ = 0.10, *p* = 0.008 | Indirect effect | -0.009 | 0.012 | -0.013 | [-0.033, 0.013] | 0.410 |
|  |  | Direct effect | -0.041 | 0.038 | -0.062 | [-0.113, 0.034] | 0.292 |
|  | UPPS sensation seeking  R^2^ = 0.13, *p* = 0.007 | Indirect effect | -0.031 | 0.021 | -0.047 | [-0.092, -0.008] | 0.019 |
|  |  | Direct effect | -0.019 | 0.037 | -0.028 | [-0.096, 0.051] | 0.578 |
|  | Sensitivity to reward  R^2^ = 0.10, *p* = 0.006 | Indirect effect | -0.014 | 0.013 | -0.021 | [-0.046, 0.006] | 0.162 |
|  |  | Direct effect | -0.036 | 0.057 | -0.054 | [-0.170, 0.051] | 0.333 |
|  |  |  |  |  |  |  |  |
| MSCEIT understanding | UPPS positive urgency  R^2^ = 0.16, *p* = 0.011 | Indirect effect | -0.032 | 0.017 | -0.051 | [-0.070, -0.005] | 0.028 |
|  |  | Direct effect | 0.027 | 0.041 | 0.044 | [-0.054, 0.111] | 0.492 |
|  | UPPS negative urgency  R^2^ = 0.10, *p* = 0.009 | Indirect effect | -0.024 | 0.013 | -0.040 | [-0.054, -0.003] | 0.024 |
|  |  | Direct effect | 0.020 | 0.042 | 0.032 | [-0.062, 0.102] | 0.638 |
|  | UPPS sensation seeking  R^2^ = 0.13, *p* = 0.010 | Indirect effect | -0.025 | 0.015 | -0.040 | [-0.058, 0.002] | 0.072 |
|  |  | Direct effect | 0.020 | 0.041 | 0.033 | [-0.063, 0.101] | 0.622 |
|  | Sensitivity to reward  R^2^ = 0.90, *p* = 0.008 | Indirect effect | -0.012 | 0.011 | -0.020 | [-0.038, 0.007] | 0.196 |
|  |  | Direct effect | 0.007 | 0.041 | 0.012 | [-0.076, 0.088] | 0.897 |
|  |  |  |  |  |  |  |  |
| MSCEIT managing | UPPS positive urgency  R^2^ = 0.16, *p* = 0.007 | Indirect effect | -0.041 | 0.017 | -0.075 | [-0.080, -0.013] | 0.001 |
|  |  | Direct effect | -0.033 | 0.034 | -0.061 | [-0.098, 0.038] | 0.289 |
|  | UPPS negative urgency  R^2^ = 0.11, *p* = 0.008 | Indirect effect | -0.024 | 0.010 | -0.044 | [-0.045, -0.006] | 0.009 |
|  |  | Direct effect | -0.050 | 0.036 | -0.093 | [-0.123, 0.020] | 0.134 |
|  | UPPS sensation seeking  R^2^ = 0.14, *p* = 0.009 | Indirect effect | -0.018 | 0.012 | -0.033 | [-0.044, 0.003] | 0.098 |
|  |  | Direct effect | -0.056 | 0.033 | -0.104 | [-0.123, 0.008] | 0.088 |
|  | Sensitivity to reward  R^2^ = 0.10, *p* = 0.007 | Indirect effect | -0.028 | 0.012 | -0.052 | [-0.057, -0.009] | 0.003 |
|  |  | Direct effect | -0.046 | 0.035 | -0.085 | [-0.116, 0.024] | 0.177 |
